# Effect modification by statin use status on the association between fine particulate matter (PM_2.5_) and cardiovascular mortality

**DOI:** 10.1093/ije/dyae084

**Published:** 2024-07-03

**Authors:** Li Bai, Jeffrey C Kwong, Jay S Kaufman, Tarik Benmarhnia, Chen Chen, Aaron van Donkelaar, Randall V Martin, JinHee Kim, Hong Lu, Richard T Burnett, Hong Chen

**Affiliations:** Primary Care & Population Health Research Program, ICES, Toronto, ON, Canada; Primary Care & Population Health Research Program, ICES, Toronto, ON, Canada; Public Health Ontario, Toronto, ON, Canada; Dalla Lana School of Public Health, University of Toronto, Toronto, ON, Canada; Department of Family and Community Medicine, University of Toronto, Toronto, ON, Canada; Department of Epidemiology and Biostatistics, McGill University, Montreal, QC, Canada; Scripps Institution of Oceanography, University of California, San Diego , La Jolla, CA, USA; Scripps Institution of Oceanography, University of California, San Diego , La Jolla, CA, USA; Department of Energy, Environment and Chemical Engineering, Washington University, St Louis, MO, USA; Department of Energy, Environment and Chemical Engineering, Washington University, St Louis, MO, USA; Public Health Ontario, Toronto, ON, Canada; Dalla Lana School of Public Health, University of Toronto, Toronto, ON, Canada; Primary Care & Population Health Research Program, ICES, Toronto, ON, Canada; Population Studies Division, Environmental Health Science and Research Bureau, Health Canada, Ottawa, ON, Canada; Primary Care & Population Health Research Program, ICES, Toronto, ON, Canada; Public Health Ontario, Toronto, ON, Canada; Dalla Lana School of Public Health, University of Toronto, Toronto, ON, Canada; Population Studies Division, Environmental Health Science and Research Bureau, Health Canada, Ottawa, ON, Canada

**Keywords:** Air pollution, cardiovascular health, statins, effect modification, mortality

## Abstract

**Background:**

Numerous studies have linked fine particulate matter (PM_2.5_) to increased cardiovascular mortality. Less is known how the PM_2.5_-cardiovascular mortality association varies by use of cardiovascular medications. This study sought to quantify effect modification by statin use status on the associations between long-term exposure to PM_2.5_ and mortality from any cardiovascular cause, coronary heart disease (CHD), and stroke.

**Methods:**

In this nested case-control study, we followed 1.2 million community-dwelling adults aged ≥66 years who lived in Ontario, Canada from 2000 through 2018. Cases were patients who died from the three causes. Each case was individually matched to up to 30 randomly selected controls using incidence density sampling. Conditional logistic regression models were used to estimate odds ratios (ORs) for the associations between PM_2.5_ and mortality. We evaluated the presence of effect modification considering both multiplicative (ratio of ORs) and additive scales (the relative excess risk due to interaction, RERI).

**Results:**

Exposure to PM_2.5_ increased the risks for cardiovascular, CHD, and stroke mortality. For all three causes of death, compared with statin users, stronger PM_2.5_-mortality associations were observed among non-users [e.g. for cardiovascular mortality corresponding to each interquartile range increase in PM_2.5_, OR = 1.042 (95% CI, 1.032–1.053) vs OR = 1.009 (95% CI, 0.996–1.022) in users, ratio of ORs = 1.033 (95% CI, 1.019–1.047), RERI = 0.039 (95% CI, 0.025–0.050)]. Among users, partially adherent users exhibited a higher risk of PM_2.5_-associated mortality than fully adherent users.

**Conclusions:**

The associations of chronic exposure to PM_2.5_ with cardiovascular and CHD mortality were stronger among statin non-users compared to users.

Key Messages

- There is limited evidence of whether cardiovascular susceptibility to air pollution differs by statin use status.
- In this large nested case-control study, we sought to quantify how the associations between long-term exposure to outdoor fine particulate matter (PM_2.5_) and mortality from any cardiovascular cause, coronary heart disease and stroke vary by use of statins.
- Following 1.2 million adults in Ontario, Canada for 19 years, we found that the effect of chronic exposure to PM_2.5_ on cardiovascular mortality was heterogenous, with more harmful effects observed among adults not using statins than statin users.
- Our findings underscore the importance of understanding heterogeneous exposure effects of PM_2.5_ across different groups of individuals, which may motivate the discovery and implementation of more specific air pollution interventions.

## Introduction

Ambient air pollution is a major concern globally.^1^ In particular, fine particulate matter ≤2.5 μm in diameter (PM_2.5_) is a leading cause of premature mortality worldwide.^2^ A large burden of PM_2.5_ on health is through its effect on the cardiovascular system^3^; biological mechanisms include cardiovascular injury by aggravating oxidative stress and systemic inflammation, accelerating atherosclerosis progression, and altering cardiac autonomic function, all of which may increase cardiovascular mortality risk.^4^ Given the ubiquitous nature of PM_2.5_ exposure and the mortality burden attributed to PM_2.5_, identifying novel strategies to complement existing air quality policies is crucial.

There has been increasing interest in heterogeneous exposure effects of PM_2.5_ across different groups of individuals, to identify population subgroups who are at higher risk and would benefit more from air pollution interventions. Recently, use of cardiovascular medications has been posited as a potential effect modifier in the association between PM_2.5_ and mortality, because medications with anti-inflammatory and antioxidant activity were repeatedly shown to modify pathological responses to air pollution.^5^ For example, statins, a widely prescribed class of cholesterol-lowering drugs, decrease C-reactive protein and other markers of inflammatory and endothelial response.^6^ Therefore, it is conceivable that statin use could modify the effect of PM_2.5_ on inflammation and heart-rate variability.^5^^,^^6^ However, epidemiological evidence as to whether cardiovascular susceptibility to air pollution differs by statin use is unclear. Several studies reported weaker associations between PM_2.5_ and circulating inflammatory markers among statin users than non-users,^7–10^ but two other studies reported no strong evidence of multiplicative effect modification by statins on the PM_2.5_-cardiovascular event association.^11^^,^^12^ Thus, whether statin non-users are at a higher risk of PM_2.5_-associated cardiovascular mortality compared to statin users remains uncertain.

The objective of this study was to evaluate whether statin use is an effect modifier in the associations of chronic PM_2.5_ exposure with cardiovascular, coronary heart disease (CHD), and stroke mortality. We also sought to determine whether the effect modification varies by statin use adherence, dosage and selected individual characteristics.

## Methods

### Study design and population

We conducted a nested case-control study among all community-dwelling adults aged ≥66 years who resided in the Canadian province of Ontario on 1 January 2000. Ontario’s residents have universal access to hospital care and physician services. Additionally, prescription drug coverage (i.e. Ontario Drug Benefit Plan, ODB) starts automatically when Ontarians reach 65 years of age. Eligible community-dwelling Ontarians (*n* = 1 171 730, exclusion criteria in the Supplementary Material, available as Supplementary data at *IJE* online) were censored when reaching the end of follow-up (31 December 2018), becoming ineligible for provincial health insurance, or death. Due to the time-dependent nature of exposures to PM_2.5_ and statin use, we applied the nested case-control design.^13^ We selected controls from the same population as the cases to minimize selection bias.^13^

### Case and control identification

Using unique encoded identifiers, we linked the study population to multiple Ontario health administrative databases and analysed them at ICES (formerly the Institute for Clinical Evaluative Sciences). We ascertained cause-specific deaths from the Office of the Registrar General Vital Statistics Death Database. We defined cases as patients who died from any cardiovascular cause, CHD, or stroke, respectively, as detailed in Supplementary Table S1, available as Supplementary data at *IJE* online.

We selected controls using incidence density sampling.^14^ Briefly, for each case, we formed a risk set of all potential controls consisting of all eligible subjects who did not die at the failure time of the cases. A random sample of up to 30 controls was selected from each risk set and matched to the case at index date (i.e. the death date) to maximize power while avoiding computational constraints.^15^ A subject was eligible to serve as a control repeatedly prior to becoming a case. Cases and controls were also matched by baseline age and sex.

### PM_2.5_ exposure assessment

We derived annual PM_2.5_ concentrations by integrating satellite-derived aerosol optical depth data with ground-level PM_2.5_ measurements, utilizing a global atmospheric chemistry transport model. This integration was refined through the application of a geographically weighted regression model.^16^ Our PM_2.5_ estimates were characterized by a spatial resolution near 1 km × 1 km and covered the period from 2000 to 2016. The model’s efficacy was validated against 2312 fixed monitoring stations in North America, exhibiting robust performance (*R*^2^ = 0.80). Owing to the limitation of data availability (2000–2016), we applied an annual calibration technique to the PM_2.5_ surfaces to align them with the study period (1997–2018), similar to previous studies (details in the Supplementary Material, available as Supplementary data at *IJE* online).^17^^,^^18^ For each year in an individual’s follow-up period, we assigned estimates of concentrations of PM_2.5_ to the individual’s annual six-character residential postal code in that year. We calculated PM_2.5_ exposure based on a 3-year rolling average, allowing for a better understanding of the long-term exposure effects of PM_2.5_.^19^

### Statin use

We identified statin use based on the ODB prescription claims database, which includes information on the date of prescription, daily dosage and number of days supplied for each prescription. Users were identified as subjects who were dispensed any statin medication within 1 year before index date (details in the Supplementary Material, available as Supplementary data at *IJE* online). We also considered statin use during different time windows (i.e. 90 days and 2 years before index date) in sensitivity analyses. We calculated daily dosing and categorized them into low-to-moderate intensity and high intensity (Supplementary Table S2, available as Supplementary data at *IJE* online), as done previously.^20^ The dose estimation in the ODB has been previously validated and found to have high accuracy (89%) in determining the daily dose of statins.^21^ Additionally, we characterized adherence by classifying users as fully adherent if days supplied reached 80% of days within 1 year before index date, and partially adherent otherwise.^22^

### Covariates

We considered various individual- and neighbourhood area-level covariates which have previously been shown to be associated with PM_2.5_ exposure and cardiovascular mortality.^23–26^ A summary of all covariates is listed in Supplementary Table S1 (available as Supplementary data at *IJE* online). Individual-level covariates included comorbidities (e.g. coronary heart disease and cancer), indicators of health-seeking behaviour (e.g. history of routine periodic health examination) and health-care use (e.g. frequencies of hospitalizations and outpatient visits). To further reduce possible confounding on the statin-mortality association, we considered procedures and treatments (e.g. prior coronary bypass grafting, dialysis) and concurrent medication use (e.g. β-blockers, loop diuretics). We used a 3-year look-back period to identify comorbidities, hospitalizations, procedures and treatment to adequately capture these long-standing health issues; a 1-year look-back period was applied to outpatient interactions and concurrent medication use which can change more frequently among the elderly.^27–29^

Additionally, to reduce confounding on the PM_2.5_-mortality association, we considered multiple time-varying contextual socioeconomic variables at the census division and dissemination levels, using 2001, 2006 and 2016 Canadian Census data (e.g. percentages of recent immigrants, population aged ≥15 years with less than high school education). As well, we created three time-varying geographic indicators of residence (northern/southern Ontario, urban/rural, and living in the Greater Toronto Area or not) based on participants’ yearly postal code to control for possible regional differences in the outcomes and statin prescriptions. We also considered duration of statin use.

### Statistical analysis

To examine the impacts of PM_2.5_ on cardiovascular, CHD, and stroke mortality, we applied conditional logistic regression models. For each outcome, we first built a basic model that included PM_2.5_ exposure (continuous), statin non-use (non-users vs users, with users as the reference level), and an interaction term between PM_2.5_ and statin non-use (details in the Supplementary Material, available as Supplementary data at *IJE* online). Odds ratios (ORs) for cardiovascular, CHD and stroke mortality were calculated per interquartile range (IQR) increase in PM_2.5_ (2.7 μg/m^3^). Sequentially adjusted models were then constructed by incrementally adding selected covariates, including duration of statin use, geographic indicators of residence, neighbourhood-level variables, comorbid conditions, variables for health-seeking behaviour, health-care utilization, history of procedures and treatments, and concurrent medication use (details in Supplementary Table S3, available as Supplementary data at *IJE* online).

We evaluated potential effect modification by statin use status in the PM_2.5_-mortality associations. We also investigated effect modification by adherence (non-users, partially adherent users vs fully adherent users) and statin dosage (non-users, low-to-moderate intensity users vs high intensity users). Additionally, we evaluated effect modification by statin use status among selected subpopulations: females, males, and patients with or without history of statin-indicated conditions (Supplementary Table S1, available as Supplementary data at *IJE* online). We quantified effect modification on both multiplicative (ratio of ORs) and additive scales (relative excess risks due to interaction, RERI) (details in the Supplementary Material, available as Supplementary data at *IJE* online).^30^ The confidence intervals of RERIs were estimated based on the ‘MOVER’ method.^31^

To test the robustness of our effect estimates, for each outcome, we conducted sensitivity analyses by further adjusting for an area-based measure used widely to represent health inequities in Ontario,^32^ using 1-year PM_2.5_ exposure, restricting the study period to 2000–2016 (when PM_2.5_ data were available), and ascertaining statin use status identified 90 days and 2 years before index date. Furthermore, given that statins might be not recommended for patients with chronic liver diseases due to the concern for hepatotoxicity,^33^ we conducted a sensitivity analysis by excluding subjects with prior chronic liver disease (Supplementary Table S1, available as Supplementary data at *IJE* online). Additionally, for cardiovascular mortality, we assessed whether the effect of PM_2.5_ in the non-users may further vary according to the presence or absence of statin-indicated conditions (which may reflect their eligibility to use statins).

## Results

### Participant characteristics

Among the study population of 1 171 730 eligible participants, 188 801 people died from any cardiovascular cause (16.1%) from 2000 to 2018 (Table 1), of which 98 445 deaths were attributable to CHD (52.1%) and 27 803 deaths were attributable to stroke (14.7%) (Supplementary Table S4, available as Supplementary data at *IJE* online). Using incidence density sampling, we matched 5 662 586, 2 952 765, and 833 974 controls for cases of cardiovascular, CHD, and stroke deaths respectively. The mean age at death from cardiovascular causes was ∼84 years. Compared with controls, cases were more likely to have more comorbidities, live in low-income neighbourhoods, use more healthcare services, and use more cardiovascular medications. The distributions of exposures to PM_2.5_ at index date were presented in Table 1 and Supplementary Table S4 (available as Supplementary data at *IJE* online).

**Table 1.** Characteristics of cases and matched controls of the cardiovascular death cohort

Characteristics [*n* (%) or mean ± standard deviation]	Controls^a^	Cases
	*N* = 5 662 586	*N* = 188 801
***Individual-level variables***		
**Demographic variables**		
Age	84.17 ± 6.86	84.19 ± 6.86
Male	2 844 592 (50.2%)	94 844 (50.2%)
Rural residence	846 468 (14.9%)	31 138 (16.5%)
Living in Northern Ontario	411 772 (7.3%)	16 128 (8.5%)
Living in greater Toronto area	2 231 257 (39.4%)	65 374 (34.6%)
**Health-seeking behaviour**		
Number of optometrist visit	0.57 ± 0.98	0.46 ± 0.88
Number of ophthalmologist visit	1.01 ± 2.17	0.83 ± 1.94
Number of cholesterol test	2.00 ± 3.07	1.74 ± 3.03
Physical examination	520 265 (9.2%)	9892 (5.2%)
Influenza vaccination	3 101 098 (54.8%)	95 925 (50.8%)
**Health care use**		
Number of hospital admissions	0.64 ± 1.47	2.32 ± 3.06
Number of primary care visits	9.25 ± 10.04	21.35 ± 20.42
Number of after-hour visits	0.17 ± 0.82	0.20 ± 1.00
Number of cardiologist visits	0.65 ± 2.18	3.08 ± 7.09
Number of neurologist visits	0.11 ± 0.77	0.53 ± 2.77
Number of mental health visits	0.26 ± 0.44	0.30 ± 0.46
Number of medications	8.66 ± 6.52	13.88 ± 8.66
Home care receipt	1 234 274 (21.8%)	102 830 (54.5%)
Rostered to the Ontario Primary Care Enrolment Models	2 666 778 (47.1%)	67 125 (35.6%)
Continuity of care		
Low	1 237 236 (21.8%)	77 695 (41.2%)
Moderate	811 552 (14.3%)	19 821 (10.5%)
High	3 613 798 (63.8%)	91 285 (48.3%)
**Comorbidities**		
Hypertension	4 234 309 (74.8%)	158 201 (83.8%)
Diabetes	1 286 110 (22.7%)	60 548 (32.1%)
Acute myocardial infarction	1 655 191 (29.2%)	112 524 (59.6%)
Congenital heart disease	34 109 (0.6%)	3 249 (1.7%)
Congestive heart failure	736 984 (13.0%)	96 916 (51.3%)
Cardiac valve disorders	135 910 (2.4%)	21 313 (11.3%)
Cardiomyopathy	26 842 (0.5%)	9480 (5.0%)
Cardiac arrhythmia	1 108 673 (19.6%)	89 855 (47.6%)
Generalized atherosclerosis	191 658 (3.4%)	16 661 (8.8%)
Other cardiovascular disorders	149 334 (2.6%)	20 453 (10.8%)
Lipid disorders	810 195 (14.3%)	27 731 (14.7%)
Chronic renal failure	291 734 (5.2%)	34 854 (18.5%)
Cancer	1 574 006 (27.8%)	57 017 (30.2%)
Cerebrovascular disease	571 445 (10.1%)	63 171 (33.5%)
Transient ischemic attack	115 330 (2.0%)	7659 (4.1%)
Emphysema, chronic bronchitis, chronic obstructive pulmonary disease	645 453 (11.4%)	46 901 (24.8%)
Dementia	501 287 (8.9%)	33 214 (17.6%)
**Procedures and treatments**		
Coronary angiography	110 291 (1.9%)	13 228 (7.0%)
Coronary bypass grafting	41 911 (0.7%)	3137 (1.7%)
Percutaneous coronary intervention	183 581 (3.2%)	17 700 (9.4%)
Peripheral bypass grafting	12 963 (0.2%)	2151 (1.1%)
Bone density test	959 244 (16.9%)	22 280 (11.8%)
Dialysis	15 984 (0.3%)	3989 (2.1%)
Chemotherapy	69 153 (1.2%)	3419 (1.8%)
**Concurrent medication use**		
Hypolipidemic agents (non-statin)	198 211 (3.5%)	7705 (4.1%)
Oral anticoagulants	557 710 (9.8%)	42 378 (22.4%)
Nitrates	709 408 (12.5%)	57 884 (30.7%)
Loop diuretics	896 896 (15.8%)	90 382 (47.9%)
Non-loop diuretics	1 114 323 (19.7%)	38 815 (20.6%)
Other antihypertensive agents	289 679 (5.1%)	12 837 (6.8%)
Antiplatelet agents	316 593 (5.6%)	21 804 (11.5%)
Angiotensin-converting enzyme inhibitors	1 862 945 (32.9%)	87 867 (46.5%)
Angiotensin receptor blockers	754 997 (13.3%)	27 568 (14.6%)
β blockers	1 544 745 (27.3%)	84 409 (44.7%)
Calcium channel blockers	1 633 207 (28.8%)	68 937 (36.5%)
Chronic obstructive pulmonary disease drugs	868 900 (15.3%)	52 586 (27.9%)
Antipsychotics	152 888 (2.7%)	17 891 (9.5%)
Antidepressants	809 517 (14.3%)	44 516 (23.6%)
Duration of the use of statin in days	511.43 ± 746.03	585.36 ± 766.14
***Area-level variables***		
Income quintiles		
Lowest	1 100 474 (19.4%)	40 619 (21.5%)
Lower middle	1 240 049 (21.9%)	42 664 (22.6%)
Middle	1 129 013 (19.9%)	37 852 (20.0%)
Upper middle	1 028 650 (18.2%)	32 792 (17.4%)
Upper	1 164 400 (20.6%)	34 874 (18.5%)
Dependency quintile^b^		
Lowest	525 960 (9.3%)	17 102 (9.1%)
Lower middle	740 028 (13.1%)	24 657 (13.1%)
Middle	967 457 (17.1%)	32 687 (17.3%)
Upper middle	1 228 238 (21.7%)	41 926 (22.2%)
Upper	2 200 903 (38.9%)	72 429 (38.4%)
Deprivation quintile^b^		
Lowest	1 048 270 (18.5%)	31 587 (16.7%)
Lower middle	1 072 406 (18.9%)	34 335 (18.2%)
Middle	1 142 043 (20.2%)	37 949 (20.1%)
Upper middle	1 223 961 (21.6%)	42 156 (22.3%)
Upper	1 175 906 (20.8%)	42 774 (22.7%)
Ethnic diversity quintile^b^		
Lowest	1 446 004 (25.5%)	51 410 (27.2%)
Lower middle	1 277 289 (22.6%)	44 559 (23.6%)
Middle	1 102 658 (19.5%)	37 145 (19.7%)
Upper middle	984 860 (17.4%)	30 821 (16.3%)
Upper	851 775 (15.0%)	24 866 (13.2%)
Instability quintile^b^		
Lowest	619 382 (10.9%)	19 307 (10.2%)
Lower middle	903 909 (16.0%)	29 376 (15.6%)
Middle	1 117 699 (19.7%)	37 580 (19.9%)
Upper middle	1 268 232 (22.4%)	43 857 (23.2%)
Upper	1 753 364 (31.0%)	58 681 (31.1%)
Percent of recent immigrants	4.17 ± 3.90	3.79 ± 3.80
Percent of population with low education^c^	25.17 ± 4.63	25.55 ± 4.61
Percent of population unemployed^d^	6.79 ± 1.30	6.80 ± 1.32
Percent of indigenous people	0.02 ± 0.04	0.03 ± 0.04
Percent of population not married	0.46 ± 0.05	0.46 ± 0.04
Percent of population with a university degree	0.28 ± 0.12	0.26 ± 0.12
Percent of visible minority	0.21 ± 0.19	0.19 ± 0.19
***PM_2.5_ exposure (μg/m^3^)*** ^e^		
Mean ± SD	8.64 ± 2.11	8.61 ± 2.20
The 25th percentile	7.30	7.13
Median	8.73	8.69
The 75th percentile	10.05	10.07

PM_2.5_, fine particulate matter with a diameter of ≤2.5 μm; SD, standard deviation.

a The control group was not unique by patient ID, because an individual was eligible to act as a control at different time intervals during follow-up before becoming a case.

b Variables used to construct the Ontario marginalization index.

c Population aged ≥15 years with under high school education.

d Population aged ≥15 years without employment.

e The 3-year moving average of exposure to PM_2.5_ at the index date.

### Main analysis

In the basic model comprising PM_2.5_, statin non-use (non-users vs users), and an interaction term of PM_2.5_ and statin non-use, the associations of PM_2.5_ with cardiovascular, CHD and stroke mortality were negative in both statin users and non-users (Supplementary Table S5, available as Supplementary data at *IJE* online). Adjustment for selected covariates strengthened the PM_2.5_-mortality associations, and the associations of PM_2.5_ with all three outcomes were positive in the fully adjusted models.

When assessing heterogenous exposure effects of PM_2.5_ by statin use status, we observed effect modification on both multiplicative [ratio of ORs = 1.033 (95% CI, 1.019–1.047)] and additive scales [RERI = 0.039 (95% CI, 0.025–0.050)] for cardiovascular mortality (Table 2). The association between PM_2.5_ and cardiovascular mortality in relation to each IQR increase in PM_2.5_ (2.7 μg/m^3^) was stronger among non-users [OR = 1.042 (95% CI, 1.032–1.053)] than users [OR = 1.009 (95% CI, 0.996–1.022)]. For CHD mortality, effect modification measures [ratio of ORs = 1.019 (95% CI, 1.001–1.038); RERI = 0.026 (95% CI, 0.005–0.038)] were slightly weaker than those for cardiovascular mortality. The OR for the association between PM_2.5_ and CHD morality was 1.050 (95% CI, 1.037–1.064) among non-users and 1.031 (95% CI, 1.013–1.048) among users. For stroke mortality, the effect of PM_2.5_ also tended to be stronger among non-users than users (ORs = 1.034 vs 1.010) with multiplicative effect modification of 1.024 (95% CI, 0.981–1.068) and additive effect modification of 0.041 (95% CI, −0.033–0.071).

**Table 2.** Effect modification by statin use status on the associations of PM_2.5_ with deaths from all cardiovascular causes, coronary heart disease, and stroke

	The association between PM_2.5_ and cause-specific mortality by statin use status					Measure of effect modification between PM_2.5_ and statin use status					
						On the multiplicative scale			On the additive scale		
	Controls	Cases	OR^a^	95% CI^a^		Ratio of ORs	95% CI		RERI	95% CI	
**Death from all cardiovascular causes**											
Users	1 893 322	73 363	1.009	0.996	1.022	Reference			Reference		
Non-users	3 769 264	115 438	1.042	1.032	1.053	1.033	1.019	1.047	0.039	0.025	0.050
**Deaths from coronary heart disease**											
Users	974 672	40 771	1.031	1.013	1.048	Reference			Reference		
Non-users	1 978 093	57 674	1.050	1.037	1.064	1.019	1.001	1.038	0.026	0.005	0.038
**Deaths from stroke**											
Users	271 606	9148	1.010	0.970	1.051	Reference			Reference		
Non-users	562 368	18 655	1.034	1.004	1.064	1.024	0.981	1.068	0.041	−0.033	0.071

PM_2.5_, fine particulate matter with a diameter of ≤2.5 μm; RERI, relative excess risks due to effect modification.

a The odds ratios (ORs) and 95% confidence intervals (CIs) for cause-specific mortality risks in relation to each interquartile range change in PM_2.5_ (2.7 μg/m^3^) using conditional logistic regression models adjusting for duration of statin use, geographic indicators (e.g. urban/rural), neighbourhood-level covariates (e.g. % of recent immigrants and population with low education), comorbidities (e.g. hypertension, diabetes, lipid disorders, chronic renal failure, cancer and dementia), health-seeking behaviours (e.g. history of physical examination and influenza vaccination), health care utilization (e.g. the numbers of hospital admissions and continuity of care), procedures and treatment (e.g. coronary bypass grafting), use of other medications (e.g. β blockers, calcium channel blockers and drugs for antipsychotics).

There were some indications that partially adherent users were at higher risk of PM_2.5_-associated mortality risk than fully adherent users (Table 3). For example, the OR for the association with cardiovascular mortality was 1.021 (95% CI, 1.001–1.042) among partially adherent users and 1.001 (95% CI, 0.986–1.016) among fully adherent users. Conversely, effect modification by statin dosage was less evident for all three outcomes (Supplementary Table S6, available as Supplementary data at *IJE* online).

**Table 3.** Effect modification by statin adherence status and effect modification by statin use status and sex on the associations of PM_2.5_ with deaths from all cardiovascular causes, coronary heart disease, and stroke

	The association between PM_2.5_ and cause-specific mortality by statin adherence/statin use status					Measure of effect modification between PM_2.5_ and statin adherence/statin use status					
						On the multiplicative scale			On the additive scale		
	Controls	Cases	OR^a^	95% CI^a^		Ratio of ORs	95% CI		RERI	95% CI	
**Death from all cardiovascular causes**											
Adherence											
Fully adherent users	1 385 871	47 102	1.001	0.986	1.016	Reference			Reference		
Partially adherent users	518 134	26 638	1.021	1.001	1.042	1.020	0.997	1.044	0.023	−0.006	0.042
Non-users	3 758 581	115 061	1.042	1.032	1.053	1.041	1.025	1.058	0.053	0.035	0.064
Females											
Users	839 210	32 100	0.998	0.978	1.017	Reference			Reference		
Non-users	1 978 784	61 857	1.045	1.031	1.059	1.047	1.026	1.069	0.053	0.029	0.066
Males											
Users	1 054 112	41 263	1.012	0.995	1.030	Reference			Reference		
Non-users	1 790 480	53 581	1.030	1.016	1.045	1.018	0.999	1.037	0.023	0.001	0.037
**Deaths from coronary heart disease**											
Adherence											
Fully adherent users	708 508	26 512	1.021	1.001	1.041	Reference			Reference		
Partially adherent users	271 915	14 475	1.047	1.020	1.074	1.025	0.995	1.057	0.032	−0.006	0.052
Non-users	1 972 342	57 458	1.050	1.036	1.064	1.028	1.007	1.050	0.041	0.016	0.055
Females											
Users	388 136	16 037	1.019	0.992	1.047	Reference			Reference		
Non-users	954 279	28 421	1.056	1.036	1.077	1.037	1.008	1.067	0.041	0.007	0.058
Males											
Users	586 536	24 734	1.030	1.008	1.053	Reference			Reference		
Non-users	1 023 814	29 253	1.035	1.016	1.054	1.004	0.981	1.028	0.011	−0.020	0.028
**Deaths from stroke**											
Adherence											
Fully adherent users	198 518	5718	1.010	0.963	1.059	Reference			Reference		
Partially adherent users	74 662	3488	1.004	0.943	1.069	0.994	0.924	1.069	−0.005	−0.145	0.029
Non-users	560 794	18 597	1.034	1.005	1.065	1.024	0.975	1.076	0.048	−0.049	0.084
Females											
Users	141 612	4904	0.985	0.931	1.042	Reference			Reference		
Non-users	336 508	11 197	1.032	0.993	1.072	1.048	0.989	1.111	0.060	−0.052	0.096
Males											
Users	129 994	4244	1.035	0.976	1.098	Reference			Reference		
Non-users	225 860	7458	1.041	0.994	1.089	1.005	0.944	1.070	0.033	−0.110	0.090

PM_2.5_, fine particulate matter with a diameter of ≤2.5 μm; RERI, relative excess risks due to effect modification.

a The odds ratios (ORs) and 95% confidence intervals (CIs) for cause-specific mortality risks in relation to each interquartile range change in PM_2.5_ (2.7 μg/m^3^) using conditional logistic regression models adjusting for duration of statin use, geographic indicators (e.g. urban/rural), neighbourhood-level covariates (e.g. % of recent immigrants and population with low education), comorbidities (e.g. hypertension, diabetes, lipid disorders, chronic renal failure, cancer and dementia), health-seeking behaviours (e.g. history of physical examination and influenza vaccination), health care utilization (e.g. the numbers of hospital admissions and continuity of care), procedures and treatment (e.g. coronary bypass grafting), use of other medications (e.g. β blockers, calcium channel blockers and drugs for antipsychotics).

Additionally, effect modification by statin use status appeared to be stronger among females than males for all three outcomes (Table 3). Furthermore, for cardiovascular and CHD mortality, effect modification was observed among those with and with no statin-indicated conditions but was only present among those with no statin-indicated conditions for stroke mortality (Supplementary Table S6, available as Supplementary data at *IJE* online).

### Sensitivity analysis

Various sensitivity analyses demonstrated that the observed effect modification by statin use status was generally robust with ratios of ORs ranging from 1.029 to 1.037 for cardiovascular mortality, 1.017 to 1.026 for CHD mortality and 1.007 to 1.025 for stroke mortality (Supplementary Table S7, available as Supplementary data at *IJE* online). Additionally, the associations between PM_2.5_ and cardiovascular mortality were similar in the non-users according to the presence or absence of statin-indicated conditions (ORs = 1.048 and 1.036) (Supplementary Table S8, available as Supplementary data at *IJE* online).

## Discussion

In this large, nested case-control study of ∼1.2 million people aged ≥66 years, PM_2.5_ pollution increased cardiovascular, CHD, and stroke mortality among both users and non-users. Particularly, we found that the adverse effects of PM_2.5_ were larger among non-users than users, implying that statin non-users are more sensitive to the harmful effects of PM_2.5_ on the cardiovascular system than users. This effect modification by statins was even stronger among females than males. Additionally, compared to fully adherent users, partially adherent users exhibited higher PM_2.5_-associated mortality risk. These findings underscore the importance of understanding the differences in effect modification by statin use status among certain patient groups, which may motivate the discovery and implementation of more specific air pollution interventions.

In our study, the PM_2.5_-mortality associations were negative in the minimally adjusted model, but increased and became positive with incremental adjustment for potential confounding variables. A similar pattern was observed in the Canadian Census Health and Environment Cohorts (CanCHEC).^34^ Additionally, the magnitude of our PM_2.5_-mortality associations based on the fully adjusted models are aligned with previous studies.^19^^,^^34–36^ For example, a cohort study of 2.4 million Canadian adults reported a hazard ratio of 1.22 (95% CI, 1.16–1.28) for cardiovascular death for each 10 μg/m^3^ increase in PM_2.5_.^19^ In a recent meta-analysis, Alexeeff *et al*.^36^ found that a 10 µg/m^3^ increase in chronic exposure to PM_2.5_ was associated with a 23% increased risk of CHD mortality and a 24% increased risk of stroke mortality. The finding of higher risks of cardiovascular mortality among non-statin users is supported by numerous randomized and observational studies.^23^^,^^37–39^

Our study extends previous knowledge by demonstrating the effect modification by statin use on the relationship between long-term exposure to PM_2.5_ and cardiovascular mortality. A handful of previous studies on short- or medium-term concentrations of air pollutants suggest that statin use may modify the cardiovascular effect of these pollutants. For example, Delfino *et al*.^9^ found that the associations between C-reactive protein and traffic PM exposures of 5 and 9 days were stronger among those not using statins than those using statins. Similarly, Alexeeff *et al*.^7^ found reduced effects of black carbon on markers of inflammatory and endothelial response among statin users compared with non-users. However, a few cohort studies of long-term exposure to PM_2.5_ did not observe effect modification by statin use.^11^^,^^12^ Of note, information on statin use was only available at baseline in these studies because the effect modification by statins was considered an exploratory analysis and thus may not correctly reflect statin use at outcome.^11^^,^^12^ In addition, those studies had limited statistical power.

The main strength of our study was a large and well-characterized cohort in Canada. With ∼1.2 million subjects and 19 years of follow-up, we had the statistical power to robustly assess the mortality risks for specific cardiovascular causes. This also offered a unique opportunity to explore the effect modification of PM_2.5_-related mortality risk by statin use status, even among specific subgroups. Another strength is precision of recording for prescribed statins and other drugs. The ODB prescription claims database contains comprehensive and highly accurate details of prescription medications dispensed to Ontario residents aged ≥65 years, which allowed us to determine ‘current’ users of statins and consider statin intensity and adherence. An additional strength was the inclusion of various personal and socioeconomic characteristics to mitigate confounding.

This study also has limitations. First, we lacked information on individual-level risk factors such as education, ethnicity, and smoking, and were unable to eliminate the possibility of residual confounding. We attempted to address this problem by including a wide range of potential confounders that are strongly associated with these personal factors, such as comorbidities, healthcare access and utilization, concurrent medication use, and neighbourhood deprivation. While this effort was beneficial to reduce confounding, especially on the statin-mortality associations, we recognize that some of these variables (e.g. comorbidities) may have fallen on the PM_2.5_-mortality pathway as mediators due to their ascertainment within the same look-back window as PM_2.5_ exposure. The adjustment for comorbidities had minimal impact on the associations between PM_2.5_ and cardiovascular and CHD mortality, suggesting that the resulting bias to these associations was relatively small. However, this adjustment had a somewhat larger impact on the PM_2.5_-stroke association. Using an indirect adjustment method, we previously found that the effects of PM_2.5_ on cardiovascular mortality and morbidity were not substantially affected by missing data on individual lifestyle and socioeconomic status, including smoking and education.^40–42^ It is also noteworthy that our estimated PM_2.5_-mortality associations are comparable with those reported in studies that had more individual data available.^43^ Second, for stroke mortality, we may have a more limited statistical power due to a relatively small number of cases. Third, our study was restricted to older adults because of data availability. However, a recent cohort study of Canadian adults aged 25–89 years reported a similar association between PM_2.5_ and cardiovascular mortality.^19^ Fourth, some patients may not have taken their prescribed statins or not taken the dose prescribed, leading to misclassification. This misclassification, if non-differential, would tend to underestimate rather than overestimate the true association. Fifth, we did not consider adverse effects of statins, such as neurocognitive effects, type 2 diabetes, and drug–drug interactions in this study.^44^ Sixth, our PM_2.5_ exposure was measured based on postal codes, which do not completely reflect personal exposure (e.g. indoor exposures, occupation-related exposures) or capture daily mobility.^45^ Lastly, our exposure data were only available for 2000–2016. Annual exposure surfaces for 1997–1999 and 2017–2018 were produced by the temporal calibration. However, our estimated associations were robust to the sensitivity analysis in which we restricted the study period to 2000–2016.

## Conclusion

In this large population-based study, compared to statin users, we observed that the effects of long-term exposure to PM_2.5_ on cardiovascular and CHD mortality were stronger among non-users. This effect modification by statin use status was less evident for the association with stroke mortality. Our finding suggests that interventions to reduce air pollution exposure might be more beneficial to statin non-users than users.

## Ethics approval

Use of the data in this study is authorized under Ontario’s privacy legislation and does not require Research Ethics Board review.

## Supplementary Material

dyae084_Supplementary_Data

## Data Availability

The dataset from this study is held securely in coded form at ICES. While legal data sharing agreements between ICES and data providers (e.g. healthcare organizations and government) prohibit ICES from making the dataset publicly available, access may be granted to those who meet pre-specified criteria for confidential access, available at www.ices.on.ca/DAS (email: das@ices.on.ca). The full dataset creation plan and underlying analytic code are available from the authors upon request, understanding that the computer programs may rely upon coding templates or macros that are unique to ICES and are therefore either inaccessible or may require modification.
