# Supplementary material for: Effect modification by statin use status on the association between fine particulate matter (PM2.5) and cardiovascular mortality
Source: Int J Epidemiol. 2024 Jul 3;53(4):dyae084. doi: 10.1093/ije/dyae084 (PMC11222296; doi:10.1093/ije/dyae084)
Supplement: dyae084_Supplementary_Data [file dyae084_supplementary_data.docx]

***Supplementary material***

**Effect modification by statin use status on the association between fine particulate matter (PM_2.5_) and cardiovascular mortality**

Bai, et al

**Table of Contents**

| Supplementary Methods. Exclusion criteria | Page 2 |
| --- | --- |
| Supplementary Methods. Annual calibration of fine particulate matter (PM_2.5_) surfaces | Page 2 |
| Supplementary Methods. Look-back windows used to identify PM_2.5_ exposure and statin use | Page 2 |
| Supplementary Methods. Interpretation of effect modification measures | Page 2 |
| Supplementary Methods. Coefficient interpretation for the interaction of PM_2.5_ and statin non-use | Page 2 |
| Table S1. Summary of variables | Page 4 |
| Table S2. Statin dosage and intensity | Page 11 |
| Table S3. Variables included in the incremental models | Page 12 |
| Table S4. Characteristics of cases and matched controls of the cohorts for coronary heart diseases (CHD) and stroke | Page 13 |
| Table S5. Associations between exposure to PM_2.5_, statin non-use, and deaths from all cardiovascular causes, coronary heart disease, and stroke | Page 16 |
| Table S6. Effect modification by statin dosage and effect modification by statin use status among those with and with no statin-indicated conditions on the associations of PM_2.5_ with deaths from all cardiovascular causes, coronary heart disease, and stroke | Page 18 |
| Table S7. Sensitivity analysis for the associations between PM_2.5_, statin non-use, and deaths from all cardiovascular causes, coronary heart disease, and stroke with the inclusion of an interaction term of PM_2.5_ and statin non-use | Page 20 |
| Table S8. Sensitivity analysis for the association between PM_2.5_ and cardiovascular mortality among all users, eligible nonusers, and ineligible nonusers | Page 22 |

**Supplementary Methods**

**Exclusion criteria**

Among all Ontarians aged ≥66 years (n=1,479,043), we applied the following exclusion criteria: (1) immigrants (n=100,805) using the Immigration, Refugee, and Citizenship Canada (IRCC) Permanent Resident Database, (2) non-residents or no health system contacts for 3 years before 1 January 2000 (n=120,471); (3) long-term care residents (n=83,339); and (4) missing PM_2.5_ exposure data and census-level data (n=2,698).

**Annual calibration of PM_2.5_ surfaces**

Since the data is only available for 2000 to 2016, we conducted annual calibration of its surfaces to relevant time periods during the study. Briefly, we rescaled the annual estimate of PM_2.5_ in 2000 to 1997–1999 by taking the ratio of the 2000 surface to the average concentration in 1991–1999 of PM2.5 at all fixed-site monitors across Ontario. Similarly, we estimated the concentrations of PM_2.5_ in 2017 and 2018 by scaling the data in 2016. We thus were able to assign annual estimates of PM_2.5_ exposure from 1997 to 2018 to the centroid of each subject's annual six-character residential postal code in that year, thereby accounting for residential mobility and long-term trends in exposure.

**Look-back windows used to identify PM_2.5_ exposure and statin use**

We used a 1-year time window for assessing statin use and a 3-year time window for assessing PM_2.5_ exposure in the main analysis, aiming to capture more recent statin use history and long-term PM_2.5_ exposure patterns. The lengths of windows also reflect our assumptions about the underlying etiologic mechanisms of the exposure and statin use.

**Interpretation of effect modification measures**

An RERI value of 0 implies no additive effect modification, whereas values greater than 0 imply super-additive (positive) effect modification and values less than 0 imply sub-additive (negative) effect modification. Similarly, a ratio of ORs of 1 implies no multiplicative effect modification, whereas ratios greater than 1 imply super-multiplicative (positive) effect modification and ratios less than 1 imply sub-multiplicative (negative) effect modification.

Super-additive effect modification means that the combined effect of PM_2.5_ and statin non-use is larger than the sum of the individual effects of the two factors, whereas super-multiplicative effect modification means that the combined effect is larger than the product of the individual effects.

Sub-additive effect modification means that the combined effect of PM_2.5_ and statin non-use is smaller than the sum of the individual effects of the two factors, whereas sub-multiplicative effect modification means that the combined effect is smaller than the product of the individual effects.

**Coefficient interpretation for the interaction of PM_2.5_ and statin non-use**

We modelled the interaction of PM_2.5_ (continuous) and statin non-use (binary, 0: users, 1: non-users). The regression equation for PM_2.5_, interacted with no statin use, is:

log(Y) = β_0_ +

+ β_1_PM

+ β_2_(statin non-use=1) + 0(statin non-use=0)

+ β_3_PM*(statin non-use=1) + 0PM*(statin non-use=0)

+ covariates

Since users (i.e., statin non-use=0) is the reference group, we have identified above with a 0 symbol rather than a β. The coefficients above have the following interpretation:

β0: intercept, log odds of outcome with baseline PM_2.5_ and statin use

β1: the change in the log odds of outcome per 10-unit increase in PM2.5 with statin use

β2: the change in the log odds of outcome associated with statin non-use vs. statin use and baseline PM_2.5_

β3: difference in slope of PM_2.5_ when statin non-use vs. statin use, or difference in effect of statin non-use vs. statin use with a 10 unit increase in PM_2.5_

exp(β0): odds of Y with baseline PM_2.5_ and statin use

exp(β1): odds ratio (OR) of Y for a 10-unit increase in PM_2.5_ and statin use vs. baseline PM_2.5_ and statin use

exp(β2): OR of Y for baseline PM_2.5_ and statin non-use vs. baseline PM_2.5_ and statin use

exp(β3): ratio of ORs [exp(β1+β3) / exp(β1)]

exp(β1+β3): OR of Y for a 10-unit increase in PM_2.5_ and statin non-use vs. baseline PM_2.5_ and statin non-use

exp(β2+β3):OR of Y for a 10-unit increase in PM_2.5_ and statin non-use vs. a 10-unit increase in PM_2.5_ and statin use

**Table S1. Summary of variables**

| **Variable** | **Data source(s) and timing** | **Definition/Code** |
| --- | --- | --- |
| **Outcomes** | | |
| Death from cardiovascular disease | ORGD (2000-2018) | ICD-9: 410-417, 420-438, 440-449; ICD-10: I20-I28, I30-I52, I60-I79 |
| Death from coronary heart disease | ORGD (2000-2018) | ICD-9: 410-414; ICD-10: I20-I25 |
| Death from stroke | ORGD (2000-2018) | ICD-9: 430, 431, 434, 436, 3623; ICD-10: I60, I61, I630-I635, I637-I64, H341 |
| **Air pollution exposure** | | |
| PM_2.5_ | 1998-2018 | 3-year moving average with 1-year lag |
| **Demographic variables** | | |
| Age | RPDB (at the index date) |  |
| Sex |  |  |
| Long-term care (LTC) residents | CCRS-LTC, ODB, OHIP, and INST (within 1 year before the index date) | Individuals in LTC are defined as patients who are found in the CCRS-LTC database or have ODB claims for individuals who are in LTC facilities or received OHIP fee codes which start with W that are coming from nursing homes or homes for aged. |
| Charlson comorbid index (CCI) | CIHI-DAD (within 3 years before the index date) | CCI is a diagnosis-based comorbidity measure which categorizes ICD diagnosis codes into pre-defined comorbid conditions, and assigns a weight ranging from 1 to 6 to each condition based on disease burden and its relative importance to patient prognosis. A total score is calculated from the sum of the weighted points, thus taking into account both the number of comorbid conditions and their seriousness. We used the algorithm developed by Deyo et al. in 1992, which includes 17 comorbid conditions. A Charlson comorbidity score of 0 refers to no comorbidity. The higher the CCI is, the sicker the patient is. |
| North/South Ontario | RPDB (at index) |  |
| Rural/Urban residents |  |  |
| Living in the GTA or not |  |  |
| **Medication use** | | |
| Statin | ODB (within 90 days, 1 year, and 2 years before index date, respectively). |  |
| Duration of statin use in days | ODB (within 5 years before index date, respectively). | For non-users, the duration is 0 day. |
| Angiotensin-converting enzyme inhibitors | ODB (within 1 year before index date) |  |
| Angiotensin receptor blockers |  |  |
| Oral anticoagulants |  |  |
| Antiplatelet agents |  |  |
| β blockers |  |  |
| Calcium channel blockers |  |  |
| Other antihypertensive agents |  |  |
| Hypolipidemic agents (non-statin) |  |  |
| Loop diuretics |  |  |
| Nitrates |  |  |
| Non-loop diuretics |  |  |
| Antipsychotics |  |  |
| Antidepressants |  |  |
| COPD drugs |  |  |
| **Health seeking behaviour and health care use** | | |
| Primary care visits | OHIP (within 1 year before the index date) | Specialty code: 00, 05 |
| Cardiologist visits |  | Specialty code: 09, 60; or Feecode: A605, A675, A606 |
| Neurologist visits |  | Specialty code: 18 |
| Mental health visits |  | Specialty code: 00; and diagnostic codes: 295, 296, 297, 298, 300, 301, 302, 306, 309, 311, 303, 304, 897, 898, 899, 900, 901, 902, 904, 905, 906, 909 |
| Optometrist visits |  | Specialty code: 56 |
| Ophthalmologist visits |  | Specialty code: 23 |
| Cholesterol test |  | Feecode: G001, L055, L066, L117, L151, L152, L153, L154, L155, L156, L243 |
| Number of hospital admissions | CIHI-DAD (within 3 years before the index date) |  |
| Number of medications | ODB (within 1 year before the index date) |  |
| Home care receipt | SAF before 2004 and HCDMOH after 2005 |  |
| Continuity of care | OHIP (within 2 years before the index date) | Continuity of care is measured by the Usual Provider Continuity (UPC) index which was used to calculate continuity using two years of OHIP data according to the formula: UPC = ni / N, where ni is the number of visits to a usual provider in a defined time period and N is the total number of visits. For a UPC index score to be calculated, a person must have made at least three primary care visits during the two-year period. Visits were restricted to those made to GP/FPs and community medicine physicians. Emergency department (ED) and inpatient visits were excluded. Low continuity refers to patients who made fewer than 50% of their visits to the same provider. Moderate and high continuity refer to patients who made 50-80% and larger than 80% of their visits to the same provider, respectively. |
| After hour visits | OHIP (within 1 year before the index date) | Feecode: Q012, A888 |
| Rostering to any of the Ontario primary care enrolment models (PEMs) | CAPE, RPDB (at the index date) | People can voluntarily enroll with a primary care physician who participates in any of the PEMs (e.g., Family Health Groups, Family Health Networks, Family Health Organizations, Comprehensive Care Models). Most patients receiving care from a GP/FP working in a PEM are enrolled with that physician. |
| **Comorbidities** | | |
| Hypertension | Ontario Hypertension Dataset (before the index date back to 1991) | A case of hypertension was defined based on ≥1 hospitalization with a hypertension diagnosis or one physician claim followed by another physician claim or hospitalization with a hypertension diagnosis within 2 years (ICD‐9 401‐405 and ICD‐10 I10‐I15). This validated algorithm has a sensitivity of 72% and a specificity of 95%. |
| Diabetes mellitus | Ontario Diabetes Database (before the index date back to 1991) | A case of diabetes was defined as an individual with ≥2 physician claims with a diabetes diagnostic code (ICD-9: 250), ≥1 drug claim for diabetes, or ≥ 1 hospitalization for diabetes within 1 year (ICD-9 code 250 and ICD‐10 codes E10‐E14). This validated algorithm has a high sensitivity (90.0%) and specificity (97.7%). |
| Coronary heart disease | Expanded Diagnosis Clusters (EDCs) of the John Hopkins Adjusted Clinical Groups (ACG) based on CIHI-DAD and OHIP (within 3 years before the index date) | EDC code: CAR03, CAR12 |
| Congenital heart disease |  | EDC code: CAR04 |
| Congestive heart failure |  | EDC code: CAR05 |
| Cardiac valve disorders |  | EDC code: CAR06 |
| Cardiomyopathy |  | EDC code: CAR07 |
| Cardiac arrhythmia |  | EDC code: CAR09 |
| Generalized atherosclerosis |  | EDC code: CAR10 |
| Cardiovascular disorders, other |  | EDC code: CAR16 |
| Emphysema, chronic bronchitis, COPD |  | EDC code: RES04 |
| Lipid disorders |  | EDC code: CAR11 |
| Chronic renal failure |  | EDC code: REN01 |
| Cancer |  | EDC code: MAL01 - MAL16, MAL18 |
| Cerebrovascular disease |  | EDC code: NUR05 |
| Chronic liver disease |  | EDC code: GAS05 |
| Transient ischemic attack |  | ICD-9: 435; ICD-10: G45, H340 |
| **Procedures and treatments** | | |
| Coronary angiography | CIHI-DAD (within 3 years before the index date) | Procedure codes: 4892, 4893, 4894, 4895, 4896, 4897, 4898, 4996, 4997; intervention codes 2HZ28, 3IP10, 3IS10 |
| Coronary bypass grafting | CIHI-DAD, OHIP (within 3 years before the index date) | Procedure codes: 481, 482; Intervention codes: 1IJ76, 1IJ80; Fee code: R742, R743 |
| Percutaneous coronary intervention | CIHI-DAD, OHIP (within 3 years before the index date) | Procedure codes: 4802, 4803, 4809; Intervention codes: 1IJ26, 1IJ50, 1IJ55, 1IJ57; Fee code: R787, R780, R797, R804, R809 |
| Peripheral bypass grafting | CIHI-DAD, OHIP (within 3 years before the index date) | Procedure codes: 5125, 5126, 5129; Intervention codes: 1KA76, 1JM76; Fee code: R787, R780, R797, R804, R809 |
| Dialysis | OHIP (within 3 years before index date) | Fee code: G860, G861, G862, G863, G864, G865, G866 |
| Bone densitometry | OHIP (within 3 years before index date) | Fee code: J854, J654, J888, J688, J856, J656, X152, X153, X149, X155 |
| Chemotherapy | OHIP (within 3 years before index date) | Fee code: G381, G281, G339, G345, G359, G075, G382, G390 |
| **Census derived variables** | | |
| Percent of recent immigrants | 2001, 2006, 2016 Canadian Census at both Census Division and Census Dissemination Area |  |
| Percent of population aged >15 years with less than high school education |  |  |
| Percent of population aged >15 years and unemployed |  |  |
| Percent of indigenous people |  |  |
| Percent of population not married |  |  |
| Percent of population with a university degree |  |  |
| Percent of visible minority |  |  |
| Income quintiles |  |  |
| Dependency quintile | ONMARG | Ontario Marginalization Index (ONMARG) is a geographically (Census) based index developed to quantify the degree of marginalization occurring across the province of Ontario. It is comprised of 4 major dimensions thought to underlie the construct of marginalization: residential instability, material deprivation, dependency and ethnic concentration. Expressed as a quintile, in comparison to all other dissemination areas that year. |
| Deprivation quintile |  |  |
| Ethnic concentration quintile |  |  |
| Instability quintile |  |  |
| **Other variables** |  |  |
| Major cardiovascular diseases | CIHI-DAD (within 3 years before the index date) | ICD-9: 39, 40, 41, 42, 430, 431, 432, 433, 434  ICD-10: I0-I7, I70-I74, I78 |
| Statin-indicated conditions | CIHI-DAD, NACRS, ODD, OHIP, OLIS (before the index date back to 1989) | myocardial infarction  ICD-9:410; ICD-10: I21  acute coronary syndromes  ICD-9: 411; ICD-10: I20.0, I24, I25  stable angina  ICD-9: 413; ICD-10: I20.1, I20.8, I20.9; OHIP: 410, 413, 412  documented coronary disease by angiography  ICD-10: I25.0, I25.1^, I25.8, I25.9; OHIP: Z442, G297; CCP: 489.2 - 489.8, 499.6, 499.7  Stroke, TIA  ICD-9: 434, 436, 435, 3623; ICD-10: G450, G451, G452, G453, G458, G459, H340, I63 (excluding I63.6), I64, H34.1; OHIP: 436, 432, 435  documented carotid disease  OHIP: N220, R792; CCI: 1JE57Lx, 1JE50x, 1JE57Gx: CCP: 5012  peripheral artery disease  ICD-9: 440.0, 440.20-440.24, 440.29, 440.9, 249.70, 249.71, 250.70-250.73, 443.81, 443.9, 444; ICD-10: I70.0, I70.2, I70.20, I70.21, I70.8, I70.9, I73.9, I74.0, I74.3, I74.4, I74.5, I74.8, I74.9, I79.2, E10.50x, E10.51x, E10.70x, E10.71x, E11.50x, E11.51x, E11.70x, E11.71x, E13.50x, E13.51x, E13.70x, E13.71x, E14.50x, E14.51x, E14.70x, E14.71x  Abdominal aortic (AAA) or previous aneurysm surgery  ICD-9: 441.3, 441.4, 441.5 and 441.6; ICD-10: I71.3, I71.4; OHIP: E627  Diabetes  ICD-9: 250; ICD‐10: E10‐E14 and drug claim for diabetes  Chronic Kidney disease  ICD-9: 4030, 4031, 4039, 4040, 4041, 4049, 585, 586, 5888, 5889, 2504; ICD-10: E102, E112, E132, E142, I12, I13, N08, N18, N19; OHIP: 403, 585  LDL-C>=5  OLIS Observationcode: 22748-8 and 39469-2  CABG  CCI: 1IJ76; CCP: 481  PCI  CCI: 1IJ50, 1IJ54, 1IJ57GQ; CCP: 4802, 4803 |

Abbreviation: CIHI-DAD = Canadian Institute for Health Information - Discharge Abstract Database; CCI = Canadian Classification of Health Interventions; CCP = Canadian Classification of Procedures; COPD = Chronic obstructive pulmonary disease; HCDMOH = Home Care Database; ICD = International Classification of Diseases; RPDB = Registered Persons Data Base; CCRS_LTC = Continuing Care Reporting System's long-term care database; INST = Institution Information System; ODB = Ontario Drug Benefit; OHIP = Ontario Health Insurance Plan; CAPE = Client Agency Program Enrolment Data; ONMARG = Ontario Marginalization Index database; OLIS = Ontario Laboratories Information System, ORGD = Ontario Registrar General information on deaths

**Table S2. Statin dosage and intensity**

| **Statin** | **Dosage** | | |
| --- | --- | --- | --- |
|  | **Low-intensity (LDL-C reduction <30%)** | **Moderate-intensity (LDL-C reduction 30% to <50%)** | **High-intensity (LDL-C reduction >50%)** |
| Atorvastatin | NA | 10 to 20 mg | 40 to 80 mg |
| Fluvastatin | 20 to 40 mg | 80 mg | NA |
| Lovastatin | 20 mg | 40 mg | NA |
| Pravastatin | 10 to 20 mg | 40 to 80 mg | NA |
| Rosuvastatin | NA | 5 to 10 mg | 20 to 40 mg |
| Simvastatin | 10 mg | 20 to 40 mg | NA |

From ACC/AHA, 2013. Dosages shown are total daily dosages.

Abbreviations: ACC=American College of Cardiology; AHA=American Heart Association; LDL-C=low-density lipoprotein cholesterol; NA=not applicable.

**Table S3. Variables included in the incremental models**

| **Model** | **Included variables** |
| --- | --- |
| Model 1 (basic model) | PM_2.5_ exposure, statin dosage, and an interaction term of PM_2.5_ and no use of statins |
| Model 2 | Further adjusted for duration of statin use |
| Model 3 | Further adjusted for urban/rural, south/north, living in Greater Toronto Area or not, and the eight neighborhood-level covariates including included % of recent immigrants, population with low education, population unemployed, indigenous people, population not married, population with a university degree, visible minority and income quintile |
| Model 4 | Further adjusted for comorbid hypertension, diabetes, acute myocardial infarction, congenital heart disease, congestive heart failure, cardiac valve disorders, cardiomyopathy cardiac arrhythmia, generalized atherosclerosis, other cardiovascular disorders, lipid disorders, chronic renal failure, cancer, cerebrovascular disease, transient ischemic attack, emphysema, chronic bronchitis, chronic obstructive pulmonary disease, and dementia |
| Model 5 | Further adjusted for numbers of optometrist visits, ophthalmologist visits, cholesterol tests, history of physical examination, influenza vaccination, numbers of hospital admissions, primary care visits, after-hour visits, cardiologist visits, neurologist visits, mental health visits, number of medications, home care receipt, rostered to the Ontario Primary Care Enrolment Models, and continuity of care |
| Model 6 | Further adjusted for history of coronary angiography, coronary bypass grafting, percutaneous coronary intervention, peripheral bypass grafting, bone density test, dialysis, and chemotherapy |
| Model 7 (main model) | Further adjusted for history of the use of hypolipidemic agents (non-statin), oral anticoagulants, nitrates, loop diuretics, non-loop diuretics, Other antihypertensive agents, Antiplatelet agents, angiotensin-converting enzyme inhibitors, angiotensin receptor blockers, β blockers, calcium channel blockers, chronic obstructive pulmonary disease drugs, antipsychotics, antidepressants |

Abbreviations: PM_2.5_ = fine particulate matter

**Table S4. Characteristics of cases and matched controls of the cohorts for coronary heart diseases (CHD) and stroke**

|  | **CHD death cohort** | | **stroke death cohort** | |
| --- | --- | --- | --- | --- |
| Characteristics [n (%) or mean ± standard deviation] | Controls | Cases | Controls | Cases |
|  | N=2,952,765 | N=98,445 | N=833,974 | N=27,803 |
| ***Individual-level variables*** |  |  |  |  |
| **Demographic variables** |  |  |  |  |
| Age | 83.38 ± 6.87 | 83.36 ± 6.88 | 84.52 ± 6.73 | 84.60 ± 6.71 |
| Male | 1,610,350 (54.5%) | 53,987 (54.8%) | 355,854 (42.7%) | 11,702 (42.1%) |
| Rural residence | 446,532 (15.1%) | 16,854 (17.1%) | 123,245 (14.8%) | 4,603 (16.6%) |
| Living in Northern Ontario | 216,858 (7.3%) | 8,730 (8.9%) | 60,131 (7.2%) | 2,284 (8.2%) |
| Living in greater Toronto area | 1,155,605 (39.1%) | 32,180 (32.7%) | 328,329 (39.4%) | 10,050 (36.1%) |
| **Health seeking behaviour** |  |  |  |  |
| Number of optometrist visit | 0.57 ± 0.96 | 0.46 ± 0.87 | 0.57 ± 0.98 | 0.46 ± 0.87 |
| Number of ophthalmologist visit | 1.00 ± 2.15 | 0.85 ± 1.95 | 1.01 ± 2.17 | 0.82 ± 1.90 |
| Number of cholesterol test | 2.03 ± 3.09 | 1.83 ± 3.11 | 1.95 ± 3.05 | 1.53 ± 2.78 |
| Physical examination | 293,625 (9.9%) | 5,417 (5.5%) | 75,997 (9.1%) | 1,660 (6.0%) |
| Influenza vaccination | 1,619,666 (54.9%) | 50,641 (51.4%) | 456,211 (54.7%) | 13,701 (49.3%) |
| **Health care use** |  |  |  |  |
| Number of hospital admissions | 0.67 ± 1.49 | 2.34 ± 3.51 | 0.63 ± 1.54 | 2.15 ± 2.03 |
| Number of primary care visits | 9.31 ± 9.98 | 20.07 ± 19.51 | 9.28 ± 10.05 | 22.22 ± 20.26 |
| Number of after-hour visits | 0.16 ± 0.78 | 0.18 ± 0.92 | 0.17 ± 0.82 | 0.19 ± 0.98 |
| Number of cardiologist visits | 0.64 ± 2.18 | 3.16 ± 7.13 | 0.63 ± 2.13 | 1.66 ± 4.18 |
| Number of neurologist visits | 0.11 ± 0.78 | 0.26 ± 1.50 | 0.10 ± 0.75 | 1.67 ± 5.20 |
| Number of mental health visits | 0.26 ± 0.44 | 0.29 ± 0.46 | 0.26 ± 0.44 | 0.32 ± 0.47 |
| Number of medications | 8.54 ± 6.45 | 14.16 ± 8.66 | 8.72 ± 6.51 | 11.52 ± 7.59 |
| Home care receipt | 602,356 (20.4%) | 49,650 (50.4%) | 189,404 (22.7%) | 14,652 (52.7%) |
| Rostered to the Ontario Primary Care Enrolment Models | 1,289,670 (43.7%) | 31,984 (32.5%) | 393,264 (47.2%) | 9,838 (35.4%) |
| Continuity of care |  |  |  |  |
| Low | 617,634 (20.9%) | 39,928 (40.6%) | 182,018 (21.8%) | 11,560 (41.6%) |
| Moderate | 426,937 (14.5%) | 10,086 (10.2%) | 119,888 (14.4%) | 3,008 (10.8%) |
| High | 1,908,194 (64.6%) | 48,431 (49.2%) | 532,068 (63.8%) | 13,235 (47.6%) |
| **Comorbidities** |  |  |  |  |
| Hypertension | 2,170,735 (73.5%) | 80,942 (82.2%) | 628,349 (75.3%) | 23,643 (85.0%) |
| Diabetes | 665,012 (22.5%) | 33,538 (34.1%) | 185,049 (22.2%) | 7,901 (28.4%) |
| Acute myocardial infarction | 887,844 (30.1%) | 70,070 (71.2%) | 239,948 (28.8%) | 11,635 (41.8%) |
| Congenital heart disease | 17,277 (0.6%) | 1,359 (1.4%) | 4,973 (0.6%) | 257 (0.9%) |
| Congestive heart failure | 379,558 (12.9%) | 53,858 (54.7%) | 109,701 (13.2%) | 8,106 (29.2%) |
| Cardiac valve disorders | 70,679 (2.4%) | 9,980 (10.1%) | 20,128 (2.4%) | 1,330 (4.8%) |
| Cardiomyopathy | 14,182 (0.5%) | 5,697 (5.8%) | 3,668 (0.4%) | 336 (1.2%) |
| Cardiac arrhythmia | 572,373 (19.4%) | 46,007 (46.7%) | 162,103 (19.4%) | 11,413 (41.0%) |
| Generalized atherosclerosis | 103,707 (3.5%) | 8,872 (9.0%) | 27,700 (3.3%) | 1,789 (6.4%) |
| Other cardiovascular disorders | 78,132 (2.6%) | 11,075 (11.2%) | 20,863 (2.5%) | 1,727 (6.2%) |
| Lipid disorders | 441,361 (14.9%) | 15,504 (15.7%) | 115,755 (13.9%) | 3,778 (13.6%) |
| Chronic renal failure | 146,705 (5.0%) | 18,324 (18.6%) | 41,824 (5.0%) | 2,814 (10.1%) |
| Cancer | 824,587 (27.9%) | 29,136 (29.6%) | 224,560 (26.9%) | 8,140 (29.3%) |
| Cerebrovascular disease | 300,519 (10.2%) | 20,629 (21.0%) | 84,189 (10.1%) | 23,909 (86.0%) |
| Transient ischemic attack | 58,845 (2.0%) | 3,648 (3.7%) | 17,092 (2.0%) | 1,537 (5.5%) |
| Emphysema, chronic bronchitis, chronic obstructive pulmonary disease | 345,689 (11.7%) | 25,780 (26.2%) | 92,636 (11.1%) | 4,863 (17.5%) |
| Dementia | 241,362 (8.2%) | 14,386 (14.6%) | 75,692 (9.1%) | 6,588 (23.7%) |
| **Procedures and treatments** |  |  |  |  |
| Coronary angiography | 59,367 (2.0%) | 9,338 (9.5%) | 15,265 (1.8%) | 703 (2.5%) |
| Coronary bypass grafting | 24,203 (0.8%) | 2,200 (2.2%) | 5,606 (0.7%) | 224 (0.8%) |
| Percutaneous coronary intervention | 99,273 (3.4%) | 12,052 (12.2%) | 25,263 (3.0%) | 999 (3.6%) |
| Peripheral bypass grafting | 7,414 (0.3%) | 949 (1.0%) | 1,775 (0.2%) | 151 (0.5%) |
| Bone density test | 487,289 (16.5%) | 10,878 (11.0%) | 153,578 (18.4%) | 3,855 (13.9%) |
| Dialysis | 8,658 (0.3%) | 2,061 (2.1%) | 2,218 (0.3%) | 245 (0.9%) |
| Chemotherapy | 35,937 (1.2%) | 1,770 (1.8%) | 9,899 (1.2%) | 448 (1.6%) |
| **Concurrent medication use** |  |  |  |  |
| Hypolipidemic agents (non-statin) | 102,146 (3.5%) | 4,368 (4.4%) | 28,477 (3.4%) | 898 (3.2%) |
| Oral anticoagulants | 293,604 (9.9%) | 21,644 (22.0%) | 81,638 (9.8%) | 4,982 (17.9%) |
| Nitrates | 377,634 (12.8%) | 38,322 (38.9%) | 105,312 (12.6%) | 4,771 (17.2%) |
| Loop diuretics | 456,064 (15.4%) | 49,765 (50.6%) | 134,701 (16.2%) | 8,095 (29.1%) |
| Non-loop diuretics | 575,296 (19.5%) | 19,718 (20.0%) | 168,541 (20.2%) | 5,549 (20.0%) |
| Other antihypertensive agents | 161,736 (5.5%) | 6,844 (7.0%) | 38,577 (4.6%) | 1,509 (5.4%) |
| Antiplatelet agents | 157,838 (5.3%) | 12,056 (12.2%) | 46,049 (5.5%) | 3,254 (11.7%) |
| Angiotensin-converting enzyme inhibitors | 983,730 (33.3%) | 49,403 (50.2%) | 272,497 (32.7%) | 11,035 (39.7%) |
| Angiotensin receptor blockers | 369,985 (12.5%) | 13,933 (14.2%) | 114,033 (13.7%) | 3,748 (13.5%) |
| β blockers | 800,071 (27.1%) | 46,523 (47.3%) | 227,562 (27.3%) | 10,431 (37.5%) |
| Calcium channel blockers | 835,707 (28.3%) | 37,253 (37.8%) | 244,576 (29.3%) | 9,284 (33.4%) |
| COPD drugs | 451,994 (15.3%) | 28,399 (28.8%) | 127,579 (15.3%) | 5,395 (19.4%) |
| Antipsychotics | 76,565 (2.6%) | 7,864 (8.0%) | 23,171 (2.8%) | 2,731 (9.8%) |
| Antidepressants | 409,150 (13.9%) | 22,559 (22.9%) | 123,558 (14.8%) | 6,319 (22.7%) |
| Duration of the use of statin in days | 495.77 ± 736.61 | 608.77 ± 770.77 | 498.36 ± 740.34 | 493.99 ± 729.94 |
| ***Area-level variables*** |  |  |  |  |
| Income quintiles |  |  |  |  |
| Lowest | 572,008 (19.4%) | 21,797 (22.1%) | 165,681 (19.9%) | 5,780 (20.8%) |
| Lower middle | 645,916 (21.9%) | 22,458 (22.8%) | 183,475 (22.0%) | 6,315 (22.7%) |
| Middle | 589,010 (19.9%) | 19,775 (20.1%) | 165,567 (19.9%) | 5,587 (20.1%) |
| Upper middle | 538,334 (18.2%) | 17,052 (17.3%) | 149,503 (17.9%) | 4,870 (17.5%) |
| Upper | 607,497 (20.6%) | 17,363 (17.6%) | 169,748 (20.4%) | 5,251 (18.9%) |
| Dependency Quintile*^b^* |  |  |  |  |
| Lowest | 277,301 (9.4%) | 8,884 (9.0%) | 76,790 (9.2%) | 2,495 (9.0%) |
| Lower middle | 386,305 (13.1%) | 12,667 (12.9%) | 108,079 (13.0%) | 3,743 (13.5%) |
| Middle | 507,811 (17.2%) | 17,172 (17.4%) | 141,736 (17.0%) | 4,731 (17.0%) |
| Upper middle | 644,587 (21.8%) | 22,091 (22.4%) | 180,411 (21.6%) | 6,125 (22.0%) |
| Upper | 1,136,761 (38.5%) | 37,631 (38.2%) | 326,958 (39.2%) | 10,709 (38.5%) |
| Deprivation Quintile***^b^*** |  |  |  |  |
| Lowest | 549,655 (18.6%) | 15,817 (16.1%) | 152,589 (18.3%) | 4,852 (17.5%) |
| Lower middle | 560,882 (19.0%) | 17,589 (17.9%) | 156,694 (18.8%) | 5,139 (18.5%) |
| Middle | 597,652 (20.2%) | 20,096 (20.4%) | 167,154 (20.0%) | 5,508 (19.8%) |
| Upper middle | 636,913 (21.6%) | 22,296 (22.6%) | 181,291 (21.7%) | 6,212 (22.3%) |
| Upper | 607,663 (20.6%) | 22,647 (23.0%) | 176,246 (21.1%) | 6,092 (21.9%) |
| Ethnic Diversity Quintile***^b^*** |  |  |  |  |
| Lowest | 764,870 (25.9%) | 27,789 (28.2%) | 211,267 (25.3%) | 7,497 (27.0%) |
| Lower middle | 668,705 (22.6%) | 23,805 (24.2%) | 187,745 (22.5%) | 6,389 (23.0%) |
| Middle | 572,306 (19.4%) | 19,077 (19.4%) | 163,263 (19.6%) | 5,591 (20.1%) |
| Upper middle | 507,452 (17.2%) | 15,512 (15.8%) | 145,787 (17.5%) | 4,595 (16.5%) |
| Upper | 439,432 (14.9%) | 12,262 (12.5%) | 125,912 (15.1%) | 3,731 (13.4%) |
| Instability Quintile*^b^* |  |  |  |  |
| Lowest | 329,582 (11.2%) | 10,097 (10.3%) | 89,084 (10.7%) | 2,911 (10.5%) |
| Lower middle | 480,596 (16.3%) | 15,604 (15.9%) | 130,007 (15.6%) | 4,310 (15.5%) |
| Middle | 589,856 (20.0%) | 19,735 (20.0%) | 162,823 (19.5%) | 5,615 (20.2%) |
| Upper middle | 659,738 (22.3%) | 22,944 (23.3%) | 187,518 (22.5%) | 6,462 (23.2%) |
| Upper | 892,993 (30.2%) | 30,065 (30.5%) | 264,542 (31.7%) | 8,505 (30.6%) |
| Percent of recent immigrants | 4.20 ± 3.95 | 3.66 ± 3.79 | 4.20 ± 3.92 | 3.92 ± 3.88 |
| Percent of population with low education***^c^*** | 25.04 ± 4.71 | 25.56 ± 4.71 | 25.10 ± 4.65 | 25.42 ± 4.59 |
| Percent of population unemployed***^d^*** | 6.74 ± 1.31 | 6.73 ± 1.33 | 6.78 ± 1.30 | 6.78 ± 1.31 |
| Percent of indigenous people | 0.02 ± 0.04 | 0.02 ± 0.04 | 0.02 ± 0.04 | 0.02 ± 0.04 |
| Percent of population not married | 0.47 ± 0.04 | 0.46 ± 0.04 | 0.46 ± 0.05 | 0.46 ± 0.04 |
| Percent of population with a university degree | 0.27 ± 0.12 | 0.25 ± 0.12 | 0.28 ± 0.12 | 0.26 ± 0.12 |
| Percent of visible minority | 0.20 ± 0.19 | 0.18 ± 0.18 | 0.21 ± 0.19 | 0.19 ± 0.19 |
| **PM_2.5_ Exposure (μg/m^3^)*^e^*** |  |  |  |  |
| Mean ± SD | 8.72 ± 2.14 | 8.69 ± 2.25 | 8.68 ± 2.11 | 8.63 ± 2.16 |
| The 25^th^ percentile | 7.33 | 7.17 | 7.33 | 7.2 |
| Median | 8.87 | 8.80 | 8.78 | 8.73 |
| The 75^th^ percentile | 10.13 | 10.20 | 10.07 | 10.07 |

Abbreviations: PM_2.5_ = fine particulate matter; COPD = Chronic obstructive pulmonary disease drugs; SD = standard deviation

***^a^***The control group was not unique by patient ID, because an individual was eligible to act as a control at different time intervals during follow-up before becoming a case

***^b^***Variables used to construct the Ontario marginalization index

***^b^***Population aged ≥15 years with under high school education

***^c^***Population aged ≥15 years without employment

***^e^***The 3-year moving avergae of exposure to PM_2.5_ at the index date

**Table S5. Associations between exposure to PM_2.5_, statin non-use, and deaths from all cardiovascular causes, coronary heart disease, and stroke**

|  | Exposure to PM_2.5_ among users | | |  | Exposure to PM_2.5_ among non-users | | |  | Statin non-use (non-users vs. users) at baseline PM_2.5_ level | | |  | The interaction term between PM_2.5_ and statin non-use (non-users vs. users) | | |
| --- | --- | --- | --- | --- | --- | --- | --- | --- | --- | --- | --- | --- | --- | --- | --- |
|  | [exp (β1)]***^a^*** | | |  | [exp (β1+ β3)]*^a^* | | |  | [exp (β2)]***^a^*** | | |  | [exp (β3)]***^a^*** | | |
| Model | OR***^b^*** | 95% CI***^b^*** | |  | OR | 95% CI | |  | OR | 95% CI | |  | Ratio of ORs | 95% CI | |
| ***Deaths from all cardiovascular causes*** | | | |  |  |  |  |  |  |  |  |  |  |  |  |
| Basic model: PM_2.5_ exposure (continuous), statin non-use (non-users vs. users), and an interaction term of PM_2.5_ × statin non-use | 0.945 | 0.936 | 0.955 |  | 0.985 | 0.977 | 0.992 |  | 0.680 | 0.653 | 0.708 |  | 1.041 | 1.029 | 1.054 |
| + duration of statin use | 0.946 | 0.936 | 0.955 |  | 0.985 | 0.977 | 0.992 |  | 0.683 | 0.653 | 0.714 |  | 1.041 | 1.029 | 1.054 |
| + geographic indicators***^c^***, and neighborhood-level covariates***^d^*** | 0.983 | 0.972 | 0.994 |  | 1.016 | 1.007 | 1.025 |  | 0.698 | 0.668 | 0.729 |  | 1.034 | 1.022 | 1.047 |
| + comorbidities***^e^*** | 0.990 | 0.979 | 1.002 |  | 1.013 | 1.004 | 1.023 |  | 1.218 | 1.164 | 1.274 |  | 1.023 | 1.010 | 1.036 |
| + health seeking behaviours***^f^*** and health care utilization***^g^*** | 1.019 | 1.006 | 1.032 |  | 1.046 | 1.036 | 1.056 |  | 1.206 | 1.149 | 1.265 |  | 1.027 | 1.013 | 1.041 |
| + procedures and treatment***^h^*** | 1.015 | 1.002 | 1.028 |  | 1.044 | 1.034 | 1.054 |  | 1.154 | 1.100 | 1.211 |  | 1.029 | 1.015 | 1.043 |
| + use of other medications***^i^*** | 1.009 | 0.996 | 1.022 |  | 1.042 | 1.032 | 1.053 |  | 1.138 | 1.084 | 1.194 |  | 1.033 | 1.019 | 1.047 |
| ***Deaths from coronary heart disease*** | | | |  |  |  |  |  |  |  |  |  |  |  |  |
| Basic model: PM_2.5_ exposure (continuous), statin non-use (non-users vs. users), and an interaction term of PM_2.5_ × statin non-use | 0.957 | 0.944 | 0.970 |  | 0.987 | 0.976 | 0.997 |  | 0.612 | 0.579 | 0.646 |  | 1.031 | 1.014 | 1.048 |
| + duration of statin use | 0.957 | 0.944 | 0.970 |  | 0.987 | 0.976 | 0.997 |  | 0.617 | 0.581 | 0.656 |  | 1.031 | 1.014 | 1.048 |
| + geographic indicators***^c^***, and neighborhood-level covariates***^d^*** | 1.005 | 0.991 | 1.020 |  | 1.028 | 1.015 | 1.040 |  | 0.635 | 0.599 | 0.673 |  | 1.022 | 1.006 | 1.039 |
| + comorbidities***^e^*** | 1.008 | 0.992 | 1.024 |  | 1.017 | 1.005 | 1.030 |  | 1.126 | 1.060 | 1.197 |  | 1.010 | 0.993 | 1.027 |
| + health seeking behaviours***^f^*** and health care utilization***^g^*** | 1.038 | 1.020 | 1.055 |  | 1.051 | 1.038 | 1.065 |  | 1.121 | 1.051 | 1.195 |  | 1.013 | 0.995 | 1.032 |
| + procedures and treatment***^h^*** | 1.037 | 1.020 | 1.054 |  | 1.050 | 1.037 | 1.064 |  | 1.106 | 1.037 | 1.180 |  | 1.013 | 0.995 | 1.031 |
| + use of other medications***^i^*** | 1.031 | 1.013 | 1.048 |  | 1.050 | 1.037 | 1.064 |  | 1.117 | 1.047 | 1.192 |  | 1.019 | 1.001 | 1.038 |
| ***Deaths from stroke*** | | |  |  |  |  |  |  |  |  |  |  |  |  |  |
| Basic model: PM_2.5_ exposure (continuous), statin non-use (non-users vs. users), and an interaction term of PM_2.5_ × statin non-use | 0.926 | 0.899 | 0.953 |  | 0.979 | 0.961 | 0.998 |  | 0.822 | 0.736 | 0.918 |  | 1.057 | 1.022 | 1.094 |
| + duration of statin use | 0.924 | 0.898 | 0.951 |  | 0.980 | 0.962 | 0.999 |  | 0.735 | 0.651 | 0.830 |  | 1.060 | 1.025 | 1.097 |
| + geographic indicators***^c^***, and neighborhood-level covariates***^d^*** | 0.955 | 0.925 | 0.986 |  | 1.006 | 0.984 | 1.029 |  | 0.750 | 0.666 | 0.845 |  | 1.054 | 1.020 | 1.089 |
| + comorbidities***^e^*** | 1.029 | 0.992 | 1.067 |  | 1.034 | 1.008 | 1.062 |  | 1.680 | 1.470 | 1.920 |  | 1.005 | 0.969 | 1.043 |
| + health seeking behaviours***^f^*** and health care utilization***^g^*** | 1.032 | 0.991 | 1.074 |  | 1.046 | 1.016 | 1.076 |  | 1.727 | 1.489 | 2.002 |  | 1.014 | 0.972 | 1.057 |
| + procedures and treatment***^h^*** | 1.025 | 0.985 | 1.067 |  | 1.043 | 1.013 | 1.073 |  | 1.648 | 1.420 | 1.912 |  | 1.017 | 0.975 | 1.060 |
| + use of other medications***^i^*** | 1.010 | 0.970 | 1.051 |  | 1.034 | 1.004 | 1.064 |  | 1.522 | 1.310 | 1.767 |  | 1.024 | 0.981 | 1.068 |

Abbreviations: PM_2.5_ = fine particulate matter

***^a^***Interpretation of the estimates is included in the section “Coefficient interpretation for the interaction of PM_2.5_ and statin non-use” above.

***^b^***The odds ratios (ORs) and 95% confidence intervals (CIs) for cause-specific mortality risks in relation to each interquartile range change in PM_2.5_ (2.7 μg /m^3^)

***^c^***Geographic indicators included urban/rural, south/north, living in the Great Toronto Area or not.

***^d^***Neighborhood-level covariates included % of recent immigrants, population with low education, population unemployed, indigenous people, population not married, population with a university degree, visible minority, and income quintiles.

***^e^***Comorbidities included hypertension, diabetes, acute myocardial infarction, congenital heart disease, congestive heart failure, cardiac valve disorders, cardiomyopathy cardiac arrhythmia, generalized atherosclerosis, other cardiovascular disorders, lipid disorders, chronic renal failure, cancer, cerebrovascular disease, chest pain, transient ischemic attack, emphysema, chronic bronchitis, chronic obstructive pulmonary disease, and dementia.

***^f^***Variables of health seeking behaviours included the numbers of optometrist visits, ophthalmologist visits, cholesterol tests, and history of physical examination and influenza vaccination.

***^g^***Variables for health care utilization included the numbers of hospital admissions, primary care visits, after-hour visits, cardiologist visits, neurologist visits, mental health visits, number of medications, home care receipt, rostered to the Ontario primary care enrolment models, and continuity of care.

***^h^***Procedures and treatment included coronary angiography, coronary bypass grafting, percutaneous coronary intervention, peripheral bypass grafting, bone density test, dialysis, and chemotherapy

***^i^***Other medications included hypolipidemic agents (non-statin), oral anticoagulants, nitrates, loop diuretics, non-loop diuretics, Other antihypertensive agents, Antiplatelet agents, angiotensin-converting enzyme inhibitors, angiotensin receptor blockers, β blockers, calcium channel blockers, drugs for chronic obstructive pulmonary disease, antipsychotics, antidepressants.

**Table S6. Effect modification by statin dosage and effect modification by statin use status among those with and with no statin-indicated conditions on the associations of PM_2.5_ with deaths from all cardiovascular causes, coronary heart disease, and stroke**

|  | The association between PM_2.5_ and cause-specific mortality by statin dosage/statin use status | | | | |  | Measure of effect modification between PM_2.5_ and statin dosage/statin use status | | | | | | |
| --- | --- | --- | --- | --- | --- | --- | --- | --- | --- | --- | --- | --- | --- |
|  |  |  |  |  |  |  | On the multiplicative scale | | |  | On the additive scale | | |
|  | Controls | Cases | OR^a^ | 95%CI***^a^*** | |  | OR | 95%CI | |  | RERI | 95%CI | |
| ***Death from all cardiovascular causes*** | |  |  |  |  |  |  |  |  |  |  |  |  |
| Dosage |  |  |  |  |  |  |  |  |  |  |  |  |  |
| High dose | 306,699 | 16,140 | 1.016 | 0.989 | 1.043 |  | Reference | | |  | Reference | | |
| Low and moderate dose | 1,586,623 | 57,223 | 1.007 | 0.993 | 1.021 |  | 0.992 | 0.964 | 1.020 |  | -0.008 | -0.045 | 0.012 |
| Non-users | 3,769,264 | 115,438 | 1.043 | 1.033 | 1.053 |  | 1.027 | 0.999 | 1.055 |  | 0.035 | 0.002 | 0.050 |
| Absence of statin-indicated conditions | | |  |  |  |  |  |  |  |  |  |  |  |
| Users | 914,171 | 23,393 | 1.009 | 0.985 | 1.033 |  | Reference | | |  | Reference | | |
| Non-users | 2,632,917 | 57,222 | 1.047 | 1.031 | 1.063 |  | 1.038 | 1.013 | 1.063 |  | 0.051 | 0.021 | 0.067 |
| Presence of statin-indicated conditions | | |  |  |  |  |  |  |  |  |  |  |  |
| Users | 979,151 | 49,970 | 1.007 | 0.991 | 1.024 |  | Reference | | |  | Reference | | |
| Non-users | 1,136,347 | 58,216 | 1.043 | 1.029 | 1.057 |  | 1.035 | 1.017 | 1.054 |  | 0.039 | 0.019 | 0.051 |
| ***Deaths from coronary heart disease*** | |  |  |  |  |  |  |  |  |  |  |  |  |
| Dosage |  |  |  |  |  |  |  |  |  |  |  |  |  |
| High dose | 149,805 | 9,114 | 1.055 | 1.019 | 1.093 |  | Reference | | |  | Reference | | |
| Low and moderate dose | 824,867 | 31,657 | 1.026 | 1.007 | 1.044 |  | 0.972 | 0.936 | 1.008 |  | -0.028 | -0.079 | -0.006 |
| Non-users | 1,978,093 | 57,674 | 1.050 | 1.037 | 1.064 |  | 0.995 | 0.961 | 1.031 |  | 0.005 | -0.042 | 0.023 |
| Absence of statin-indicated conditions | | |  |  |  |  |  |  |  |  |  |  |  |
| Users | 454,047 | 10,873 | 1.027 | 0.993 | 1.062 |  | Reference | | |  | Reference | | |
| Non-users | 1,366,431 | 26,085 | 1.050 | 1.027 | 1.072 |  | 1.022 | 0.987 | 1.058 |  | 0.033 | -0.014 | 0.053 |
| Presence of statin-indicated conditions | | |  |  |  |  |  |  |  |  |  |  |  |
| Users | 520,625 | 29,898 | 1.030 | 1.009 | 1.052 |  | Reference | | |  | Reference | | |
| Non-users | 611,662 | 31,589 | 1.056 | 1.037 | 1.076 |  | 1.025 | 1.001 | 1.049 |  | 0.029 | 0.002 | 0.044 |
| ***Deaths from stroke*** |  |  |  |  |  |  |  |  |  |  |  |  |  |
| Dosage |  |  |  |  |  |  |  |  |  |  |  |  |  |
| High dose | 42,901 | 1,774 | 1.046 | 0.957 | 1.143 |  | Reference | | |  | Reference | | |
| Low and moderate dose | 228,705 | 7,374 | 1.000 | 0.958 | 1.044 |  | 0.956 | 0.871 | 1.051 |  | -0.046 | -0.256 | -0.015 |
| Non-users | 562,368 | 18,655 | 1.034 | 1.005 | 1.065 |  | 0.989 | 0.904 | 1.082 |  | 0.016 | -0.216 | 0.106 |
| Absence of statin-indicated conditions | | |  |  |  |  |  |  |  |  |  |  |  |
| Users | 133,524 | 3,542 | 0.959 | 0.890 | 1.034 |  | Reference | | |  | Reference | | |
| Non-users | 394,801 | 9,499 | 1.066 | 1.016 | 1.120 |  | 1.112 | 1.029 | 1.200 |  | 0.133 | -0.022 | 0.202 |
| Presence of statin-indicated conditions | | |  |  |  |  |  |  |  |  |  |  |  |
| Users | 138,082 | 5,606 | 1.016 | 0.959 | 1.077 |  | Reference | | |  | Reference | | |
| Non-users | 167,567 | 9,156 | 1.014 | 0.970 | 1.059 |  | 0.997 | 0.937 | 1.061 |  | 0.003 | -0.130 | 0.045 |

Abbreviations: PM_2.5_ = fine particulate matter; RERI = relative excess risks due to interaction

*^a^*The odds ratios (ORs) and 95% confidence intervals (CIs) for cause-specific mortality risks in relation to each interquartile range change in PM_2.5_ (2.7 μg /m^3^) using conditional logistic regression models adjusting for duration of statin use, geographic indicators (e.g., urban/rural), neighborhood-level covariates (e.g., % of recent immigrants and population with low education), comorbidities (e.g., hypertension, diabetes, lipid disorders, chronic renal failure, cancer, and dementia), health seeking behaviours (e.g., history of physical examination and influenza vaccination), health care utilization (e.g., the numbers of hospital admissions and continuity of care), procedures and treatment (e.g., coronary bypass grafting), use of other medications (e.g., β blockers, calcium channel blockers, and drugs for antipsychotics).

**Table S7. Sensitivity analysis for the associations between PM_2.5_, statin non-use, and deaths from all cardiovascular causes, coronary heart disease, and stroke with the inclusion of an interaction term of PM_2.5_ and statin non-use**

|  | Exposure to PM_2.5_ among users | | |  | Exposure to PM_2.5_ among non-users | | |  | Statin non-use (non-users vs. users) at baseline PM_2.5_ level | | |  | The interaction term between PM_2.5_ and Statin non-use (non-users vs. users) | | |
| --- | --- | --- | --- | --- | --- | --- | --- | --- | --- | --- | --- | --- | --- | --- | --- |
|  | [exp (β1)]***^a^*** | | |  | [exp (β1+ β3)]***^a^*** | | |  | [exp (β2)]***^a^*** | | |  | [exp (β3)]***^a^*** | | |
|  | OR***^b^*** | 95% CI***^b^*** | |  | OR | 95% CI | |  | OR | 95% CI | |  | Ratio of OR | 95% CI | |
| ***Death from all cardiovascular causes*** |  |  |  |  |  |  |  |  |  |  |  |  |  |  |  |
| Main model***^c^*** | 1.009 | 0.996 | 1.022 |  | 1.042 | 1.032 | 1.053 |  | 1.138 | 1.084 | 1.194 |  | 1.033 | 1.019 | 1.047 |
| Main model further adjusted for an area-level measure of health inequities***^d^*** | 1.011 | 0.998 | 1.024 |  | 1.045 | 1.035 | 1.055 |  | 1.136 | 1.082 | 1.192 |  | 1.034 | 1.020 | 1.048 |
| Excluding people with chronic liver diseases | 1.010 | 0.997 | 1.023 |  | 1.042 | 1.032 | 1.053 |  | 1.143 | 1.089 | 1.200 |  | 1.032 | 1.018 | 1.046 |
| Using statin use within 2 years before index | 1.007 | 0.995 | 1.020 |  | 1.045 | 1.034 | 1.055 |  | 1.147 | 1.093 | 1.204 |  | 1.037 | 1.023 | 1.051 |
| Using statin use within 90 dyas before index | 1.004 | 0.990 | 1.018 |  | 1.040 | 1.030 | 1.050 |  | 1.281 | 1.220 | 1.346 |  | 1.036 | 1.021 | 1.051 |
| Using 1-year PM_2.5_ exposure | 1.004 | 0.990 | 1.017 |  | 1.040 | 1.029 | 1.051 |  | 1.122 | 1.067 | 1.179 |  | 1.036 | 1.022 | 1.051 |
| Restricted the study period to 2010-2016 | 1.014 | 1.001 | 1.028 |  | 1.044 | 1.034 | 1.054 |  | 1.145 | 1.088 | 1.204 |  | 1.029 | 1.015 | 1.044 |
| ***Deaths from coronary heart disease*** |  |  |  |  |  |  |  |  |  |  |  |  |  |  |  |
| Main model***^c^*** | 1.031 | 1.013 | 1.048 |  | 1.050 | 1.037 | 1.064 |  | 1.117 | 1.047 | 1.192 |  | 1.019 | 1.001 | 1.038 |
| Main model further adjusted for an area-level measure of health inequities***^d^*** | 1.033 | 1.016 | 1.051 |  | 1.054 | 1.040 | 1.068 |  | 1.116 | 1.046 | 1.191 |  | 1.020 | 1.002 | 1.038 |
| Excluding people with chronic liver diseases | 1.033 | 1.016 | 1.051 |  | 1.051 | 1.037 | 1.065 |  | 1.126 | 1.054 | 1.202 |  | 1.017 | 0.999 | 1.036 |
| Using statin use within 2 years before index | 1.028 | 1.011 | 1.045 |  | 1.053 | 1.039 | 1.067 |  | 1.105 | 1.035 | 1.179 |  | 1.024 | 1.006 | 1.043 |
| Using statin use within 90 dyas before index | 1.024 | 1.005 | 1.043 |  | 1.050 | 1.037 | 1.063 |  | 1.226 | 1.148 | 1.309 |  | 1.026 | 1.006 | 1.045 |
| Using 1-year PM_2.5_ exposure | 1.026 | 1.008 | 1.044 |  | 1.046 | 1.031 | 1.060 |  | 1.114 | 1.043 | 1.191 |  | 1.020 | 1.001 | 1.039 |
| Restricted the study period to 2010-2016 | 1.035 | 1.018 | 1.053 |  | 1.050 | 1.036 | 1.064 |  | 1.135 | 1.061 | 1.214 |  | 1.014 | 0.995 | 1.033 |
| ***Deaths from stroke*** |  |  |  |  |  |  |  |  |  |  |  |  |  |  |  |
| Main model***^c^*** | 1.010 | 0.970 | 1.051 |  | 1.034 | 1.004 | 1.064 |  | 1.522 | 1.310 | 1.767 |  | 1.024 | 0.981 | 1.068 |
| Main model further adjusted for an area-level measure of health inequities***^d^*** | 1.012 | 0.972 | 1.054 |  | 1.037 | 1.008 | 1.068 |  | 1.517 | 1.306 | 1.763 |  | 1.025 | 0.983 | 1.070 |
| Excluding people with chronic liver diseases | 1.008 | 0.968 | 1.050 |  | 1.034 | 1.004 | 1.064 |  | 1.519 | 1.307 | 1.767 |  | 1.025 | 0.982 | 1.069 |
| Using statin use within 2 years before index | 1.019 | 0.980 | 1.060 |  | 1.031 | 1.001 | 1.061 |  | 1.688 | 1.454 | 1.959 |  | 1.012 | 0.970 | 1.054 |
| Using statin use within 90 dyas before index | 1.021 | 0.976 | 1.067 |  | 1.028 | 1.000 | 1.057 |  | 1.693 | 1.449 | 1.977 |  | 1.007 | 0.962 | 1.054 |
| Using 1-year PM_2.5_ exposure | 1.006 | 0.964 | 1.049 |  | 1.024 | 0.993 | 1.056 |  | 1.536 | 1.318 | 1.790 |  | 1.018 | 0.975 | 1.063 |
| Restricted the study period to 2010-2016 | 1.017 | 0.975 | 1.060 |  | 1.034 | 1.005 | 1.065 |  | 1.538 | 1.315 | 1.798 |  | 1.018 | 0.974 | 1.063 |

Abbreviations: PM_2.5_ = fine particulate matter

***^a^***Interpretation of the estimates is included in the section “Coefficient interpretation for the interaction of PM_2.5_ and statin non-use” above.

***^b^***The odds ratios (ORs) and 95% confidence intervals (CIs) for cause-specific mortality risks in relation to each interquartile range change in PM_2.5_ (2.7 μg /m^3^)

***^c^***Adjusted for duration of statin use, geographic indicators (e.g., urban/rural), neighborhood-level covariates (e.g., % of recent immigrants and population with low education), comorbidities (e.g., hypertension, diabetes, lipid disorders, chronic renal failure, cancer, and dementia), health seeking behaviours (e.g., history of physical examination and influenza vaccination), health care utilization (e.g., the numbers of hospital admissions and continuity of care), procedures and treatment (e.g., coronary bypass grafting), use of other medications (e.g., β blockers, calcium channel blockers, and drugs for antipsychotics).

***^d^***The Ontario Marginalization Index (i.e., dependency, deprivation, ethnic diversity, and instability)

**Table S8. Sensitivity analysis for the association between PM_2.5_ and cardiovascular mortality among all users, eligible nonusers, and ineligible nonusers**

|  |  | OR***^a^*** | 95% CI***^a^*** | |
| --- | --- | --- | --- | --- |
| **Main model** | | |  |  |
| All users |  | 1.009 | 0.996 | 1.022 |
| All nonusers |  | 1.042 | 1.032 | 1.053 |
| **Reclassifying the non-users into subcategories of eligible and not eligible nonusers*^b^*** | | | | |
| All users |  | 1.009 | 0.997 | 1.022 |
| Non-users |  |  |  |  |
| Eligible for statin |  | 1.048 | 1.035 | 1.061 |
| Ineligible for statin |  | 1.036 | 1.023 | 1.049 |

Abbreviations: PM_2.5_ = fine particulate matter

*^a^*The odds ratios (ORs) and 95% confidence intervals (CIs) for cause-specific mortality risks in relation to each interquartile range change in PM_2.5_ (2.7 μg /m^3^) using conditional logistic regression models adjusting for duration of statin use, geographic indicators (e.g., urban/rural), neighborhood-level covariates (e.g., % of recent immigrants and population with low education), comorbidities (e.g., hypertension, diabetes, lipid disorders, chronic renal failure, cancer, and dementia), health seeking behaviours (e.g., history of physical examination and influenza vaccination), health care utilization (e.g., the numbers of hospital admissions and continuity of care), procedures and treatment (e.g., coronary bypass grafting), use of other medications (e.g., β blockers, calcium channel blockers, and drugs for antipsychotics).

***^b^***Eligible and not eligible nonusers were classified by the presence or absence of statin-indicated conditions (please see eTable1 for definitions)
